# Online Rapid Detection Method of Fertilizer Solution Information Based on Characteristic Frequency Response Features

**DOI:** 10.3390/s23031116

**Published:** 2023-01-18

**Authors:** Jianian Li, Yuan Gao, Jingyuan Zeng, Xing Li, Zhuoyuan Wu, Guoxuan Wang

**Affiliations:** 1 Faculty of Modern Agricultural Engineering, Kunming University of Science and Technology, Kunming 650500, China; 2 Guangdong Provincial Key Laboratory of Conservation and Precision Utilization of Characteristic Agricultural Resources in Mountainous Areas, College of Computer Science, Jiaying University, Meizhou 514015, China

**Keywords:** dielectric properties, fertilizer sensor, characteristic frequency, frequency response features, type identification, concentration detection

## Abstract

Online rapid detection of a fertilizer solution’s type and concentration is crucial for intelligent water and fertilizer machines to realize intellectual precision variable fertilization. In this paper, a cylindrical capacitance sensor was designed based on the dielectric properties of the fertilizer solution, and an online rapid detection method of fertilizer type and concentration was proposed based on the characteristic frequency response mode. Three fertilizer solutions, potassium chloride, calcium superphosphate, and urea, were used as test objects. Ten concentrations of each fertilizer solution in the 10~100 g/L range were taken as the test fertilizer solution. Then, under the action of a series of sine wave excitation signals from 1 kHz to 10 MHz, the sensor’s amplitude-frequency/phase-frequency response data were obtained. The detection strategy of ‘first type, then concentration’ was adopted to realize rapid online detection of fertilizer type and concentration. Experimental results indicated that the maximum relative error of the sensor stability test was 0.72%, and the maximum error of concentration detection was 7.26%. Thus, the intelligent water and fertilizer machine can give feedback on the information of a fertilizer solution in real-time during the process of precise variable fertilization, thus improving the intelligence of water and fertilizer machines.

## 1. Introduction

As a new type of agricultural technology, integrating water and fertilizer can improve the utilization of chemical fertilizer, improve crop yield and quality, reduce agricultural production costs, and alleviate environmental pollution. Countries worldwide are rapidly developing and actively promoting the integration of water and fertilizer technology [1,2]. With the development of information technology, the current water and fertilizer integration technology mainly relies on intelligent water and fertilizer machines to inject fertilizer into the irrigation system to realize precise variable fertilization of crops. Rapid online detection of fertilizer type and concentration information is the key to realizing precise variable fertilization of intelligent water and fertilizer machines. In addition, it is a challenge in this research field. Therefore, it is of great practical significance to study the online rapid detection method of fertilizer information to improve the intelligence of water and fertilizer machines and overcome the technical difficulties in this field.

Currently, the methods for detecting fertilizer information mainly include the EC (Electric Conductivity)/pH (hydrogen ion concentration) detection method and the ion-selective electrode method. The EC/pH detection method usually uses the conductivity sensor and the pH sensor to measure the fertilizer solution’s EC value and pH value. Then, it analyzes the real-time changes of the nutrient ion components in the fertilizer solution and uses precise control technology to make the fertilizer solution quickly reach the expected EC/pH value, thus meeting the moisture and nutrients required by the crop [3,4,5]. This method is used by the market’s mainstream intelligent water and fertilizer machines. For example, the NetaJet irrigation system for greenhouses from Natefim, Israel, the NetaFlex irrigation system for soilless cultivation, the FertKit irrigation system for field orchards, and the Nutri-Line irrigation and fertilization system from Priva, Netherlands, all use the EC/pH online detection method to detect fertilizer solution information [6,7]. The ion-selective electrode method mainly uses the ion-selective electrode and the reference electrode to form a pair of detection electrodes. The measuring instrument measures the potential difference between the ion-selective and reference electrodes. Then, the activity or concentration of the target ion in the nutrient solution is calculated [8]. This method has been applied to real-time detection of ion concentration in the nutrient solution. For example, a real-time nutrient solution detection system for hydroponic plants was developed using NO_3_^−^, NH_4_^+^, K^+^, Ca^2+^, and Mg^2+^ ion-selective electrodes, which can effectively monitor the concentration changes of nitrate nitrogen and metal ions in hydroponic solution [9,10,11]. Nutrient solution formulations were prepared using NO_3_^−^, K^+^, and Ca^2+^ ion selective electrodes for five crops, including Yamazaki lettuce, Yamazaki strawberry, Yamazaki tomato, Cornell lettuce, and Cornell strawberry [12,13,14].

However, the EC/pH detection method can only reflect the fertilizer solution’s total nutrient ion concentration and pH value. It cannot reflect the concentration of a specific ion in the fertilizer solution [15,16,17]. Although the ion-selective electrode method can detect and analyze the concentration of a specific ion in the fertilizer solution, the detection of each specific ion requires a corresponding ion-selective electrode [18]. Moreover, the ion selective electrode suffers from the problems of cross-sensitivity, temperature drift, and response time lag [19], and there are no mature detection electrodes for some of the macro-elements in the fertilizer solution (H_2_PO_4_^−^, SO_4_^2−^, etc.) [20]. Thus, the above two methods have inherent limitations, and there is still a significant gap in achieving online rapid and accurate detection of fertilizer types and concentrations. For other detection methods of a solution, conductivity/spectrophotometry can be used to detect the concentration of sodium, potassium, magnesium, calcium, and chloride ions in artificial freshwater [21]. The pulse photoacoustic effect can be used to detect the content of methylene blue in dye [22]. Machine vision analysis can detect the content of target substances in complex mixtures [23]. Light transmission methods can realize online detection of drug concentration [24]. The UV fluorescence method can be used to detect the content of oil in freshwater online [25]. However, the detection time of these methods is too long, and the detection effect could be more stable. It cannot be well combined with the specific process of irrigation and fertilization applications. Therefore, this paper proposed a characteristic frequency response characteristic method based on the dielectric properties of a fertilizer solution. Through testing the dielectric frequency response data for urea, potassium chloride, and calcium superphosphate, this paper investigated the excitation signal characteristic frequency used to identify the type of fertilizer solution and detect the concentration of the fertilizer solution, constructed its characteristic frequency response mode, and developed the detection method and strategy. In this way, rapid identification and accurate detection of the type and concentration of a fertilizer solution can be realized. Meanwhile, during the automatic mixing of fertilizer or precise variable fertilization, the intelligent water and fertilizer machine can give feedback on the information of a fertilizer solution in real-time, thus achieving precise irrigation and fertilization according to the preset water and fertilizer ratio and further improving its intelligence.

## 2. Fertilizer Detection Principle

As a dielectric medium composed of water and water-soluble fertilizer, the nutrient components reflect its specific characteristics in the form of ions. These nutrient ions are polarized under the action of an external alternating electric field, and the electric field exists inside the fertilizer solution so that the fertilizer solution shows conductivity [26]. When the fertilizer solution is placed between a capacitor plate and an alternating electric field applied externally, the fertilizer solution will continue to polarize and move from one polarization state to another new polarization state. In this case, the polarization of the fertilizer solution lags behind the change of the external alternating electric field, leading to the fertilizer solution’s electrical energy consumption and dielectric loss. As a result, there will be a phase difference between the electric field inside the fertilizer solution and the external alternating electric field [27,28]. According to the dielectric properties of the fertilizer solution, when the frequency of the external alternating electric field is different, the phase difference will also be different, which is the phase–frequency response characteristics of the fertilizer solution.

The dielectric constant of the fertilizer solution is a comprehensive parameter that reflects the variety and concentration of the fertilizer solution, and there is a correspondence between the dielectric constant and the variety and concentration of the water-soluble fertilizer [29]. The dielectric constant of the fertilizer solution will also change when the type and concentration of nutrient ions in the fertilizer solution change. Under the action of the alternating electric field, the dielectric constant of fertilizer ε_r_ is a complex number, and its imaginary part can be expressed as a function of the frequency of the external alternating electric field, which the capacitance and conductance between the capacitor plate and the fertilizer can obtain [30] as:

(1) $$\varepsilon_{r}{}^{\prime \prime }=\frac{G}{2\pi fC_{0}}$$

where ε_r_″ is the imaginary part of the dielectric constant, C_0_ is the dielectric constant in vacuum (8.85 × 10^−12^ F/m), G is the conductance (S) between the capacitor plate after adding fertilizer solution, and f is the frequency of the external alternating electric field (Hz).

It can be seen from Formula (1) that when the overall structure of the capacitor and the fertilizer solution is unchanged, the conductance G between the capacitor plates is constant. The dielectric constant of the fertilizer solution is only affected by the external alternating electric field frequency f, i.e., the response characteristics of the fertilizer solution to the external alternating electric field mainly depend on the frequency of the electric field. When the fertilizer solution is placed between capacitor plates, the capacitance size C of the capacitor can be expressed as:

(2) $$C=\frac{\varepsilon_{r}\cdot S}{4\pi kd}$$

where ε_r_ is the dielectric constant of the fertilizer solution, S is the direct area of the capacitor plate (m^2^), d is the distance between capacitor plates (m), and k is the electrostatic force constant (8.987551 × 10^9^ Nm^2^/C^2^).

It can be seen from Formula (2) that when the dielectric constant of the fertilizer solution changes, the capacitance value of the capacitor will also change. Depending on the external alternating electric field, the capacitor will continue to charge and discharge, and the discharge voltage is closely related to the capacitance value [31]. According to the dielectric properties of the fertilizer, when the frequency of the external alternating electric field changes, the dielectric constant of the fertilizer will change, and the capacitance value of the capacitor will also change. Consequently, the amplitude of the capacitor discharge voltage changes [32], which is called the amplitude–frequency response characteristics of the fertilizer solution.

Different types and concentrations of nutrient ions in the frequency range of 1 kHz~10 MHz have a unique frequency response mode [33,34]. Therefore, using the dielectric frequency response method, the fertilizer solution is placed in the capacitor, and an excitation signal of 1 kHz~10 MHz is applied. According to the response data of the amplitude–frequency/phase–frequency of the fertilizer solution, it is feasible to construct the fertilizer solution’s amplitude–frequency/phase–frequency response mode and then realize the detection of the type and concentration of the fertilizer solution.

## 3. Fertilizer Detection Sensor Design

### 3.1. Sensor Structure Design

**Figure 1:**
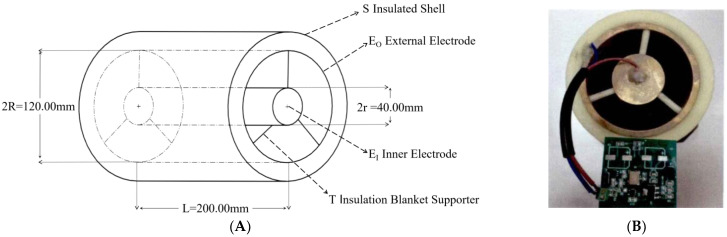
Fertilizer solution detection sensor. (**A**) The structural diagram. (**B**) The physical map.

To adapt to the irrigation pipeline and enable the sensor to detect the fertilizer liquid in the pipeline in real-time without affecting the flow of the fertilizer liquid in the pipeline, the sensor designed in this paper used a cylindrical ring structure, as shown in Figure 1 [35]. The sensor mainly consists of an outer electrode E_O_ made of copper and an inner electrode E_I_. The diameter of the outer electrode E_O_ is 120.00 mm, the diameter of the inner electrode E_I_ is 40.00 mm, and the length of the sensor L is 200.00 mm. E_O_ and E_I_ are fixed using an insulation bracket T. The outer surface of E_O_ is covered with a nylon insulation shell S to reduce the influence of the external environment on the measurement accuracy and sensitivity of the sensor. Meanwhile, after installing a pipeline interface at both ends of the sensor, it can be connected with the main pipeline of irrigation and fertilization to realize online detection in the pipeline. According to the theory of dielectric constant in electromagnetism, the specific parameters of the fertilizer sensor in this study are substituted when the sensor is filled with a dielectric constant of ε_r_. The theoretical capacitance is represented as [36]:

(3) $$C=\frac{Q}{\Delta U}=\frac{2\pi \varepsilon_{0}\varepsilon_{r}L}{\ln\frac{R}{r}}=1.0123\times 10^{-11}\cdot \varepsilon_{r}$$

where ε_0_ is the vacuum dielectric constant (8.85 × 10^−12^ F/m), ε_r_ is the dielectric constant of the fertilizer to be tested, L is electrode length of 200.00 mm, R is the outer electrode radius of 60.00 mm, and r is the inner electrode radius of 20.00 mm.

It can be seen from Formula (3) that when the structural parameters of the fertilizer detection sensor are fixed, the equivalent capacitance C of the sensor is only related to the dielectric constant ε_r_ of the fertilizer to be measured. Meanwhile, when the type and concentration of nutrient ions in the fertilizer solution to be measured in the sensor are different, the dielectric constant ε_r_ of the fertilizer solution to be measured is different, so the equivalent capacitance C of the sensor also varies.

### 3.2. Signal Conditioning Circuit Design of the Sensor

**Figure 2:**
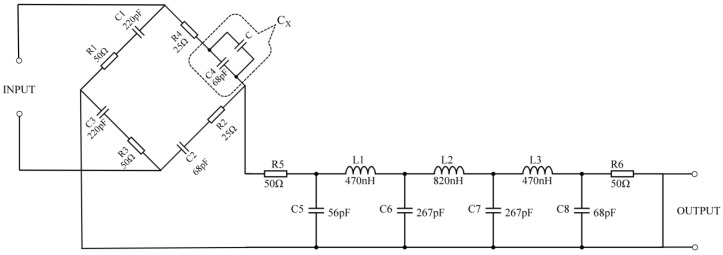
The sensor signal conditioning circuit.

The fertilizer changes in the output signal are relatively small to be measured using the fertilizer detection sensor. In addition, the capacitive sensor has high output impedance, making it susceptible to parasitic capacitance and causing the output signal to have harmonics. Therefore, designing the corresponding signal conditioning circuit for the fertilizer detection sensor is necessary. The resistance–capacitance AC bridge circuit has high sensitivity and accuracy regarding inductive capacitance change and capacitive conversion [37], and its applicable frequency range of the AC signal is 1 Hz~100 MHz [38,39]. It can well meet the detection requirements of the dielectric frequency response method proposed in this paper. Therefore, this paper uses the resistance–capacitance AC bridge and LC filter circuits to design the signal conditioning circuit, as shown in Figure 2.

The fertilizer detection sensor is connected in parallel with the capacitance C_4_. The capacitance of the sensor is equivalent to capacitance C, and the capacitance C_4_ is C_X_. Then, C_X_ and resistance R_4_ are connected in series to form a bridge arm of the resistance–capacitance AC bridge. The balance condition of resistance capacitance alternating current bridge is expressed as:

(4) $$\frac{R_{1}+j\omega C_{1}}{R_{3}+j\omega C_{3}}=\frac{R_{4}+j\omega C_{4}}{R_{2}+j\omega C_{2}}$$

When there is no fertilizer to be tested in the sensor, the bridge is in equilibrium, and the output voltage is zero when R_1_ = R_3_ = 50 Ω, C_1_ = C_3_ = 220 pF, R_2_ = R_4_ = 25 Ω, and C_2_ = C_4_ = 68 pF; when the sensor is filled with the fertilizer to be measured, the equivalent capacitance C of the sensor changes, and then the capacitance value of C_X_ changes, which causes the bridge to be in an unbalanced state. Suppose a sine wave excitation signal is applied to the input end of the conditioning circuit. In that case, the excitation signal will act on the sensor and periodically charge and discharge the capacitor C_X_, thereby generating a non-zero voltage signal at the output end. When the nutrient composition and concentration of the fertilizer to be measured in the sensor are different, the dielectric constant of the fertilizer will be different. As a result, the capacitance C_X_ varies so that the energy released by the sensor is different, which will cause the amplitude and phase of the output voltage signal to change accordingly.

When the fertilizer detection sensor contains different types and concentrations of fertilizer and the sensor is excited by different frequency signals, the equivalent capacitance C of the sensor will change. Since the fertilizer detection sensor supports different response modes to the excitation signal, it will output different amplitude–frequency/phase–frequency response data. Following a sine wave excitation signal of 1 kHz~10 MHz to the sensor with different kinds and concentrations of fertilizer, the sensor measures the amplitude–frequency/phase–frequency response data of the output and obtains the frequency response mode of different kinds and concentrations of fertilizer. By analyzing the response mode, the fertilizer solution’s type identification and concentration detection can be realized.

## 4. Test Materials and Methods

### 4.1. Test Materials and Instruments

In this paper, three types of water-soluble elemental fertilizers are commonly used in agricultural production, namely, potassium chloride (KCl) fertilizer, superphosphate (SSP) fertilizer, and urea fertilizer, are used as experimental objects. They are abbreviated as the K fertilizer, P fertilizer, and N fertilizer, respectively. The potassium content in the potassium chloride fertilizer was 60%, the phosphorus in the calcium superphosphate fertilizer was 45%, and the nitrogen content in the urea fertilizer was 46.7%.

The test instruments used in this paper include: (1) a self-developed fertilizer detection sensor to hold the fertilizer under testing for testing and sensing fertilizer type and concentration changes; (2) a function signal generator (model KEYSIGHT 33500B) to provide a 1 kHz~10 MHz sine wave excitation signal for sensor dielectric frequency detection; and (3) a digital oscilloscope (model Tektronix MD03104) to measure the perceptible voltage, waveform amplitude, phase difference, and other dielectric frequency parameters of the fertilizer detection sensor.

### 4.2. Test Method

**Figure 3:**
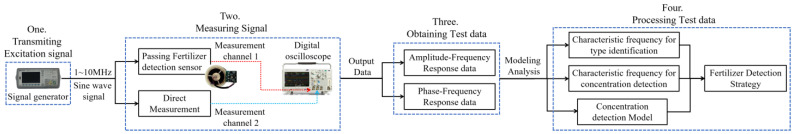
The schematic diagram of the test.

The test method and process of this paper are shown in Figure 3. Firstly, the prepared fertilizer to be tested is placed in the designed fertilizer detection sensor. Then, a series of sine wave excitation signals with different frequencies (the amplitude is 24 V, and the initial phase is 0 degree) is output by the function signal generator and applied to the input end of the fertilizer detection sensor. Subsequently, measurement channel 1 of the digital oscilloscope is connected to the output end of the fertilizer detection sensor. After the excitation signal is acted on the fertilizer to be tested, the amplitude and phase of the signal are measured, and the excitation signal output by the function signal generator is monitored in real-time using the measurement channel 2 of the digital oscilloscope. Therefore, the digital oscilloscope can measure the phase difference of the excitation signal passing through the fertilizer detection sensor. Finally, under the 1 kHz~10 MHz sine wave excitation signal, according to the amplitude–frequency/phase–frequency data measured in the experiment, the characteristic frequency, the frequency response mode, and the characteristic frequency and detection model for fertilizer concentration detection for identifying the three fertilizers are analyzed and determined. Based on this, the strategy of fertilizer type identification and concentration detection is proposed.

The fertilizer solution to be tested is prepared using a constant volume method. A specific mass of fertilizer is dissolved in a corresponding volume of deionized water according to the concentration ratio. The range of fertilization concentration in agricultural production is 10~100 g/L. In this study, potassium chloride, calcium superphosphate, and urea fertilizer solutions were prepared into ten types of concentrations of fertilizer solution to be tested: 10 g/L, 20 g/L, 30 g/L, 40 g/L, 50 g/L, 60 g/L, 70 g/L, 80 g/L, 90 g/L, and 100 g/L. To facilitate experimental analysis, the frequency range of 1 kHz~10 MHz is divided into three frequency bands: 1 kHz~100 kHz, 100 kHz~1000 kHz, and 1 MHz~10 MHz. Ten frequency points are evenly selected for each frequency band, obtaining 30 frequency points. Then, different concentrations of fertilizer solution were tested for each type, and 30 frequency points were applied to the excitation signal to test the dielectric frequency characteristics. In this way, 900 sets of amplitude–frequency/phase–frequency response data were obtained.

Due to the different types and concentrations of fertilizer solutions, the 1 kHz~10 MHz frequency range has a unique frequency response mode. That is, under the same or different excitation signals, the amplitude–frequency/phase–frequency mode of different fertilizer solutions will be different. Therefore, by comprehensively analyzing the 900 sets of amplitude–frequency/phase–frequency response data, the frequency response mode of a particular fertilizer solution can be determined to distinguish it from the other two frequency response modes. The frequency point corresponding to the frequency response mode is used as the characteristic frequency excitation signal for detecting the fertilizer solution. Then, 300 sets of amplitude–frequency response data corresponding to the potassium chloride fertilizer solution, calcium superphosphate fertilizer solution, and urea fertilizer solution were comprehensively analyzed. Following the principle of a significant one-way relationship between concentration and amplitude, the characteristic frequency excitation signal for concentration detection was determined. Based on this, a concentration detection model was constructed.

To minimize systematic error and random error in the test, the inner cavity of the sensor should be filled with the fertilizer solution to be tested, and the test should be repeated five times, with the mean value as the test result. Additionally, the stability test of the sensor is required before the dielectric frequency response characteristics are tested to determine whether the sensor has good stability during the detection process. Agricultural production in a single irrigation and fertilization operation is usually completed within 2–3 h, and among the three types of fertilizer solution to be tested, urea has a certain corrosive. Therefore, this paper uses the urea solution as the test object of the sensor stability test, configures urea solutions of 10 concentrations (10~100 g/L), and places them in the sensor for continuous testing for 5 h. In this test, the amplitude–frequency/phase–frequency response data are recorded every 30 min.

## 5. Test Results and Analysis

### 5.1. Fertilizer Detection Sensor Stability Test

**Figure 4:**
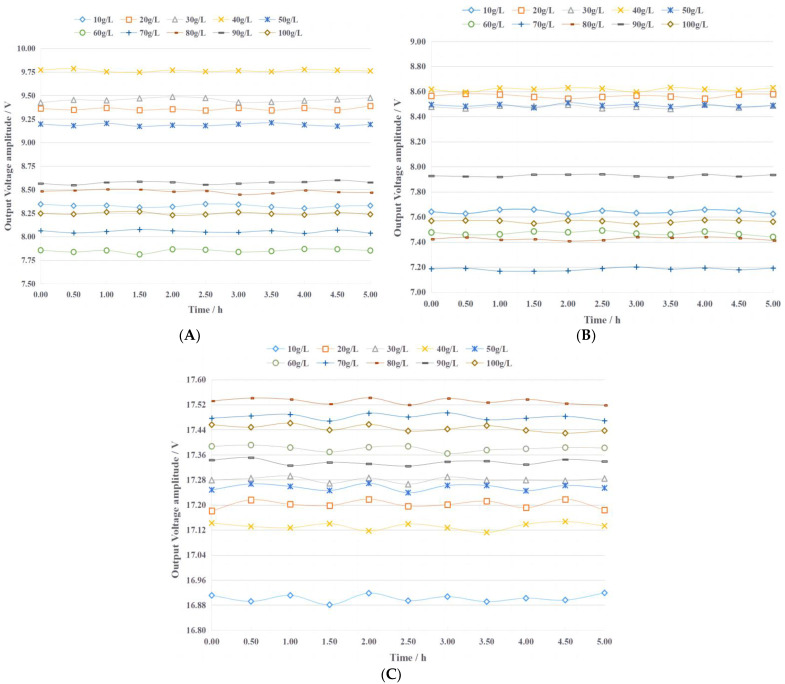
The stability test results of the fertilizer detection sensor under different frequency excitation signals. (**A**) 50 kHz (**B**) 500 kHz (**C**) 5000 kHz.

In the frequency range of 1 kHz~10 M Hz, three excitation signals of low frequency (50 kHz), medium frequency (500 kHz), and high frequency (5 MHz) were selected and applied to the fertilizer detection sensor to test its stability. For each excitation signal, the output voltage of the sensor containing ten concentrations of urea fertilizer was tested, and the results are presented in Figure 4. The output voltage amplitude level of the sensor is greatly affected by the concentration of the urea fertilizer solution and the excitation signal. For the sensor containing a particular concentration of urea fertilizer solution under the action of a specific excitation signal, the output signal voltage remains relatively stable within 5 h. The maximum relative errors under the excitation signals of 50 kHz, 500 kHz, and 5 MHz are 0.72%, 0.68%, and 0.22%, respectively. This shows that the fertilizer detection sensor has good stability and can meet the requirements of actual agricultural production.

### 5.2. Determination of Characteristic Frequency of Fertilizer Type Identification

**Figure 5:**
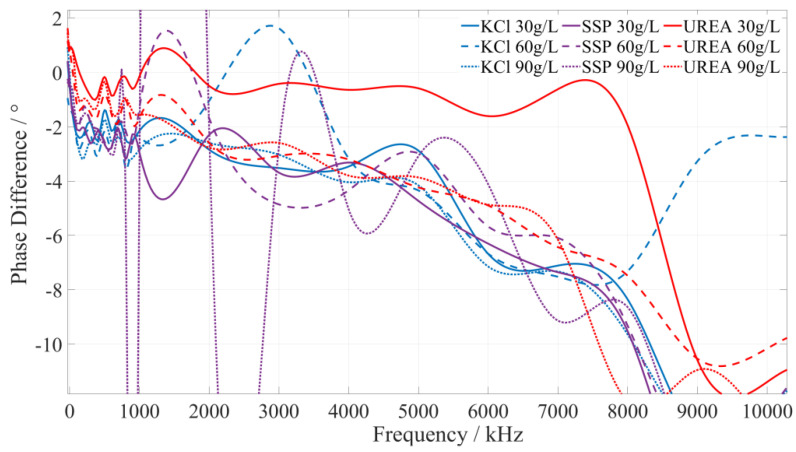
The phase–frequency characteristic curve of the potassium chloride, superphosphate, and urea fertilizer solutions under the excitation signal of 1 kHz~10 MHz.

Using the pre-test, it was found that the type of fertilizer solution is related to its phase–frequency characteristics. Under the same or different frequency excitation signals, the measured phase differences of different fertilizer solutions are different, i.e., they have different phase–frequency response modes. Although the specific value is different, different concentrations of the same fertilizer phase–frequency response mode generally have a consistent trend for the 1 kHz~10 MHz excitation signal. To reduce the amount of data processing and facilitate the presentation of test data, for the phase–frequency response data obtained from the test of the urea, potassium chloride, and calcium superphosphate fertilizer solution, the phase–frequency response data of 30 g/L, 60 g/L, and 90 g/L were further extracted. Then, the phase–frequency response data of the three types of fertilizer solutions were fitted and analyzed using Matlab, and the phase–frequency characteristics of the fertilizer solution are shown in Figure 5.

#### 5.2.1. Identify the Characteristic Frequency of Potassium Chloride (KCl) Fertilizer

**Figure 6:**
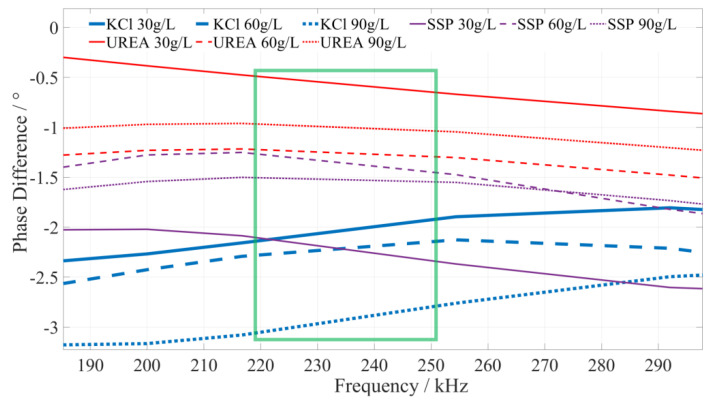
The phase–frequency characteristic curve of the potassium chloride, urea, and superphosphate fertilizer solutions under the excitation signal of 220~250 kHz.

Based on a comprehensive analysis of the phase-frequency characteristics shown in Figure 5, the phase–frequency response mode of the potassium chloride fertilizer solution under the excitation signal of 220~250 kHz (the interval is shown in the green box in Figure 6) is significantly different from that of the urea and calcium superphosphate fertilizer solution. The phase difference between the response and excitation signals of the potassium chloride fertilizer solution shows a monotonically increasing trend. However, the phase difference in the concentration of the urea and calcium superphosphate fertilizer solution monotonically decreases. To facilitate the rapid identification of the fertilizer solution, we selected 225 kHz, 235 kHz, and 245 kHz as characteristic frequencies to identify the type of potassium chloride fertilizer solution. When the excitation signals of the three characteristic frequencies are applied to a particular fertilizer solution, if the phase difference shows a monotonically increasing trend, it can be determined that this fertilizer solution is a potassium chloride fertilizer solution.

#### 5.2.2. Identify the Characteristic Frequency of Calcium Superphosphate (SSP) Fertilizer

**Figure 7:**
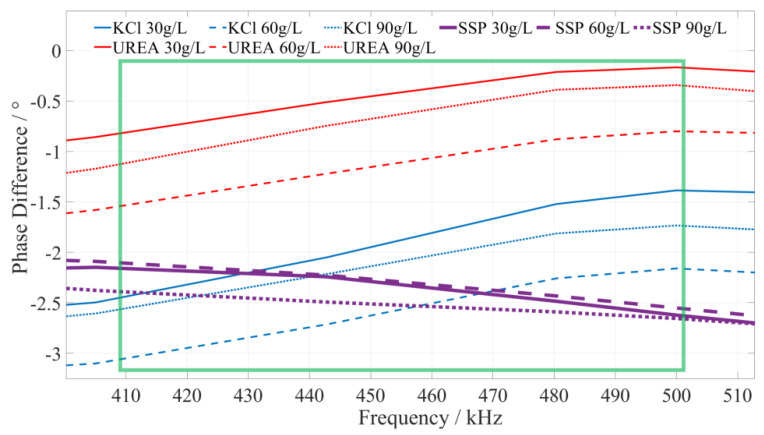
The phase–frequency characteristic curve of superphosphate, urea, and potassium chloride fertilizer solutions under the excitation signal of 410~500 kHz.

Based on a comprehensive analysis of the phase–frequency characteristics in Figure 5, the phase–frequency response mode of the calcium superphosphate fertilizer solution under the excitation signal of 410~500 kHz (the interval is shown in the green box in Figure 7) is significantly different from that of the urea and potassium chloride fertilizer solution. The phase difference between the response signal and the excitation signal of the calcium superphosphate fertilizer solution gradually decreases. However, the phase difference in the concentration of the urea and potassium chloride fertilizer solution monotonically increases. To facilitate the rapid identification of fertilizer, we selected 420 kHz, 455 kHz, and 490 kHz as characteristic frequencies to identify the type of potassium chloride fertilizer. When the excitation signals of the three characteristic frequencies are applied to a particular fertilizer, if the detected phase difference shows a monotonically decreasing trend, it can be determined that this fertilizer solution is a calcium superphosphate fertilizer solution.

#### 5.2.3. Identify the Characteristic Frequency of Urea Fertilizer

**Figure 8:**
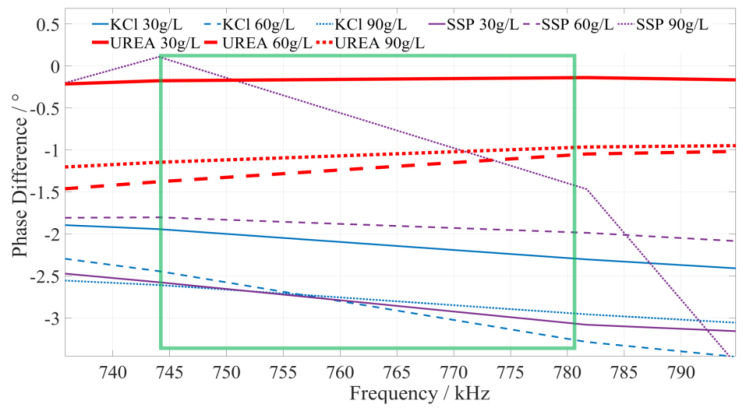
The phase–frequency characteristic curve of urea, potassium chloride, and superphosphate fertilizer solutions under the excitation signal of 745~780 kHz.

Based on a comprehensive analysis of the phase–frequency characteristics shown in Figure 5, the phase–frequency response mode of the urea fertilizer solution under the excitation signal of 745~780 kHz (the interval is shown in the green box in Figure 8) is significantly different from that of the calcium superphosphate and potassium chloride fertilizer solution. The phase difference between the urea fertilizer solution’s response and excitation signal gradually increases. However, the phase difference between the concentration of potassium chloride and calcium superphosphate fertilizer solution gradually decreases. To facilitate the rapid identification of fertilizer solution, we selected 750 kHz, 760 kHz, and 770 kHz as characteristic frequencies to identify the types of urea fertilizer solution. When the excitation signals of the three types of characteristic frequencies are applied to a particular fertilizer solution, if the detected phase difference shows a monotonically increasing trend, it is determined that this fertilizer solution is a urea fertilizer solution.

### 5.3. Determination of Characteristic Frequency of Fertilizer Concentration Detection

**Figure 9:**
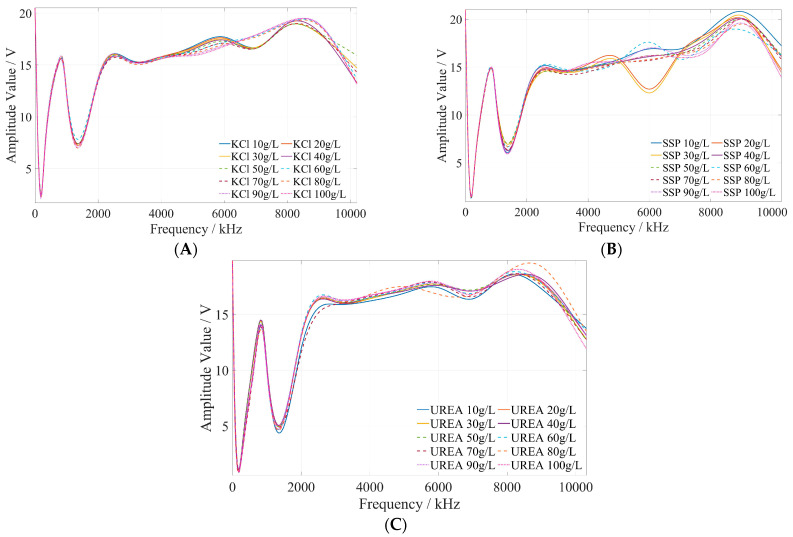
The amplitude–frequency characteristic curve of potassium chloride, superphosphate, and urea fertilizer solutions under the excitation signal of 1 kHz~10 MHz. (**A**) Potassium chloride fertilizer solution. (**B**) Calcium superphosphate solution. (**C**) Urea fertilizer solution.

The preliminary test and analysis results indicate that the fertilizer concentration is related to its amplitude–frequency characteristics. Then, the amplitude–frequency response data of potassium chloride, calcium superphosphate, and urea fertilizer were fitted and analyzed using Matlab. The amplitude–frequency characteristics of fertilizer are illustrated in Figure 9. It can be seen that the same fertilizer solution with different concentrations has different amplitude responses under the excitation signal of the same frequency, and the same fertilizer solution has different amplitude responses under the excitation signals of different frequencies.

**Figure 10:**
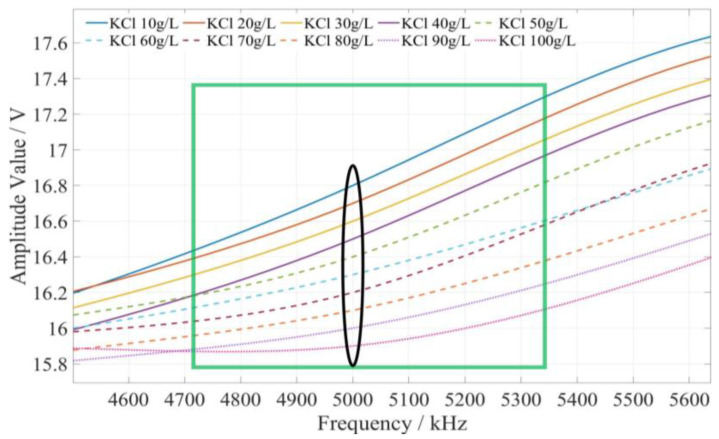
The amplitude–frequency characteristic curve of the KCl fertilizer solution under the excitation signal of 4710~5430 kHz.

**Figure 11:**
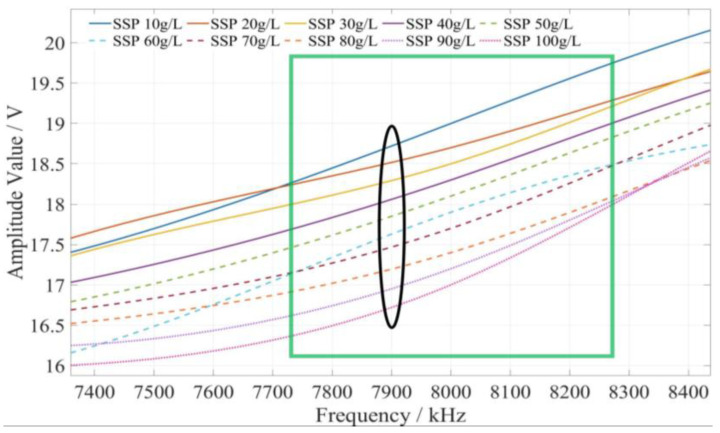
The amplitude–frequency characteristic curve of the SSP fertilizer solution under the excitation signal of 7740~8280 kHz.

**Figure 12:**
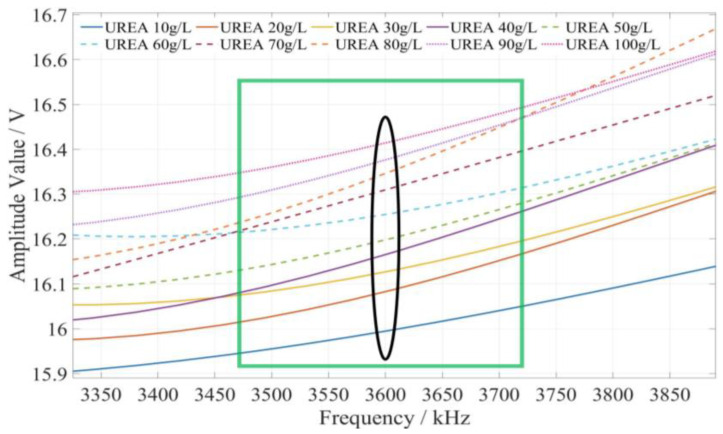
The amplitude–frequency characteristic curve of the urea fertilizer solution under the excitation signal of 3470~3720 kHz.

The method of determining the characteristic frequency for fertilizer concentration detection is described as follows. Based on the comprehensive analysis of the amplitude–frequency response characteristics of the three types of fertilizer solutions in Figure 9, for each fertilizer solution, the frequency band of the excitation signal with monotonic characteristics of the fertilizer solution and its response amplitude is first searched under the excitation signal of 1 kHz~10 MHz. In this frequency band, the frequency of the excitation signal with the most uniform interval between the response amplitudes of each fertilizer solution is selected as the characteristic frequency to detect the concentration of the fertilizer solution. Based on the above methods and analysis results, it is found that the amplitude–frequency characteristics of different concentrations of the potassium chloride fertilizer solution decrease monotonically only in the frequency range of 4710~5430 kHz (as shown in the green box in Figure 10). The response amplitude of each concentration is the most uniform at 5000 kHz (as shown in the black ellipse in Figure 10). Meanwhile, the amplitude–frequency characteristics of different concentrations of the calcium superphosphate fertilizer solution decrease monotonically only in the frequency range of 7740~8280 kHz (as shown in the green box in Figure 11). The response amplitude of each concentration is the most uniform at 7900 kHz (as shown in the black ellipse in Figure 11). In addition, the amplitude–frequency characteristics of different concentrations of the urea fertilizer solution decrease monotonically only in the frequency range of 3470~3720 kHz (as shown in the green box in Figure 12). The response amplitude of each concentration is the most uniform at 3600 kHz (as shown in the black ellipse in Figure 12). Therefore, the excitation signals of 5000 kHz, 7900 kHz, and 3600 kHz can be selected as the characteristic frequency excitation signals to detect the concentrations of potassium chloride, calcium superphosphate, and urea.

### 5.4. Establishment of the Fertilizer Concentration Detection Model

**Figure 13:**
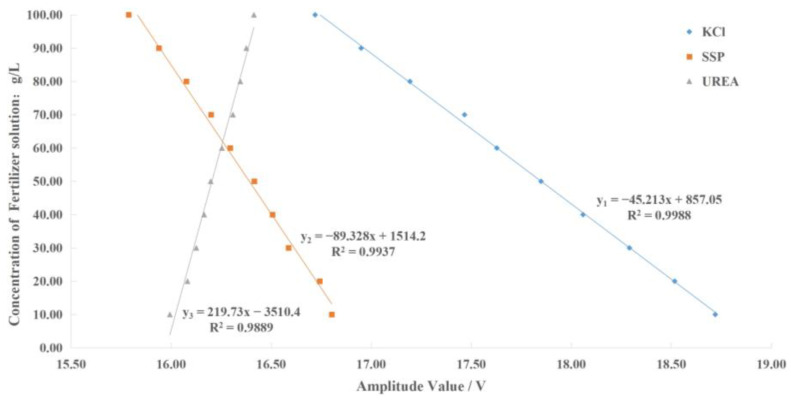
The relationship between fertilizer solution concentration and amplitude.

The amplitude–frequency response data of different concentrations of the potassium chloride, calcium superphosphate, and urea fertilizer solution under the excitation signal of the corresponding concentration detection characteristic frequency were fitted using Matlab. Then, the fertilizer concentration detection model was constructed as shown in Figure 13, where *x*_1_, *x*_2_, and *x*_3_ represent the output signal voltage amplitude of the sensor under the excitation signal of the characteristic frequency (5000 kHz, 7900 kHz, and 3600 kHz) of potassium chloride, calcium superphosphate, and urea fertilizer, respectively; *y*_1_, *y*_2_, and *y*_3_ represent the concentration detection values of potassium chloride, calcium superphosphate, and urea fertilizer solution, respectively.

### 5.5. Fertilizer Solution Type Identification and Concentration Detection Methods and Strategies

**Figure 14:**
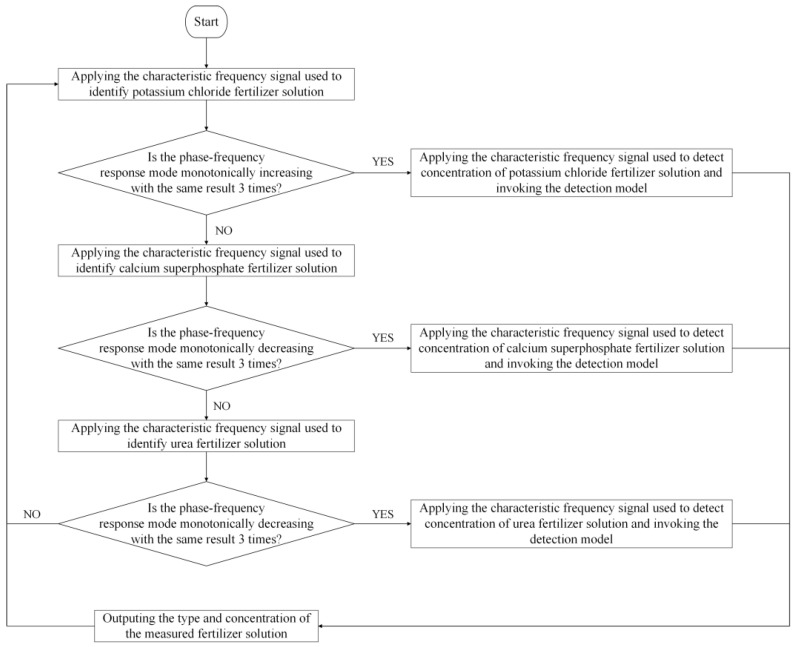
Identification of fertilizer solution type and its concentration detection strategy.

According to the characteristic frequency excitation signal and its characteristic frequency response mode, the strategy of ‘first type and then concentration’ is adopted to detect the information of the fertilizer solution. First, identify the type of fertilizer solution and then detect the concentration of the fertilizer solution. The specific detection strategy and process are presented in Figure 14. When the fertilizer detection sensor is used to detect the fertilizer under testing, the identification of fertilizer solution type and concentration detection is carried out in the order of potassium chloride, calcium superphosphate, and urea.

To identify the type of fertilizer solution as potassium chloride and detect its concentration, we chose the characteristic frequency excitation signals of 225 kHz, 235 kHz, and 245 kHz for identifying the potassium chloride fertilizer solution applied to the sensor. Then, the phase–frequency response data of the sensor are obtained under the excitation signal of these three characteristic frequencies, and whether the result is consistent with the characteristic frequency response mode of identifying potassium chloride is determined. If it is consistent, the current fertilizer solution under testing is potassium chloride. To avoid the misjudgment caused by the even error of the sensor response signal, we adopted the identification strategy of redundant judgment, i.e., the determination of the characteristic frequency response mode is repeated three times. If there are more than two times of consistency with the characteristic frequency response mode of potassium chloride, the current fertilizer solution under testing is a potassium chloride solution. Then, the characteristic frequency (5000 kHz) excitation signal is applied to the sensor to detect the concentration of the potassium chloride fertilizer solution. According to the amplitude–frequency response data output by the sensor, the concentration detection model of the potassium chloride fertilizer solution constructed above is employed to calculate the current concentration of the potassium chloride fertilizer solution. When the detection result is not potassium chloride, the same method is applied to identify the type and concentration of calcium superphosphate. If the detection result is still not calcium superphosphate, the same method is applied to identify the type and concentration of urea.

### 5.6. Application Verification of the Fertilizer Concentration Detection Model

To verify the effectiveness of the detection model, we prepared a series of different concentrations of fertilizer solutions by mixing urea, potassium chloride, and calcium superphosphate with deionized water in the concentration range of 5~90 g/L at a room temperature of 25 °C. Each fertilizer solution was configured with 14 concentration gradients, each concentration of each fertilizer solution was tested three times, and the interval between each test was 1 h. During the test, the characteristic frequency excitation signals of 7900 kHz, 5000 kHz, and 3600 kHz were applied to the potassium chloride, calcium superphosphate, and urea fertilizer solution for concentration detection, respectively. Then, the amplitude of the output signal of the sensor was measured using a digital oscilloscope, and the amplitude was substituted into the fertilizer concentration detection model to verify the accuracy of the fertilizer concentration detection model. The test results are presented in Table 1.

The test results indicate that the maximum error of the detection model is 7.26%, which can meet the application requirements of irrigation and fertilization systems for online detection of fertilizer concentration in agricultural engineering.

## 6. Conclusions

(1) To realize the rapid online detection of fertilizer type and concentration and real-time feedback of fertilizer information for intelligent water and fertilizer machines in automatic mixing or precise variable fertilization, this paper designed a cylindrical capacitance sensor for detecting fertilizer information. It proposed an online rapid detection method of fertilizer information based on the characteristic frequency response mode.
(2) The stability of the sensor was tested. The RMS of the output signal voltage remained relatively stable within 5 h, and the maximum relative errors under the excitation signals of 50 kHz, 500 kHz, and 5 MHz were 0.72%, 0.68%, and 0.22%, respectively. The results indicated that the sensor has good stability and can meet the requirements of actual agricultural production.
(3) The sensor’s phase–frequency and amplitude–frequency response data were tested under the sine wave excitation signal of 1 kHz~10 MHz. The analysis results indicated that the phase–frequency response characteristics of potassium chloride, calcium superphosphate, and urea fertilizer are related to the fertilizer type, and the amplitude–frequency response characteristics are related to the fertilizer concentration. Specifically, the characteristic frequencies of the potassium chloride fertilizer solution are 225 kHz, 235 kHz, and 245 kHz. The characteristic frequencies of the superphosphate fertilizer solution are 420 kHz, 455 kHz, and 490 kHz. The characteristic frequencies of the urea fertilizer solution are 750 kHz, 760 kHz, and 770 kHz. The characteristic frequencies of potassium chloride, calcium superphosphate, and urea are 5000 kHz, 7900 kHz, and 3600 kHz, respectively. Finally, three fertilizer concentration detection models were constructed with R^2^ greater than 0.9889.
(4) According to the determined characteristic frequency and its characteristic frequency response mode, a detection strategy of ‘first type, then concentration’ is proposed. The effectiveness of the proposed method was verified with 14 concentrations of potassium chloride, calcium superphosphate, and urea fertilizer samples. The maximum relative error was 7.26%, indicating that the rapid online detection of fertilizer type and concentration can be realized.
(5) Compared with EC/pH detection and the ion-selective electrode method, the conductivity sensor, pH sensor, and ion-selective electrode will be affected by the coupling of ion type and temperature but also indirectly detect fertilizer concentration. The online rapid detection method of fertilizer solution information based on the characteristic frequency response mode proposed in this paper can detect the specific concentration of nitrogen, phosphorus, and potassium ions in the fertilizer solution. At the same time, the cylindrical capacitance sensor designed in this paper based on the dielectric characteristics of the fertilizer solution does not have cross-sensitivity, temperature drift, and response time lag. It can directly identify the type of fertilizer liquid and detect the concentration of the fertilizer liquid. It is suitable for fertilizer liquid irrigation and can further improve the intelligence of precision variable fertilization.

## Figures and Tables

**Table 1:** The verification results of the detection model.

Actual Concentration (g/L)	Urea		Potassium Chloride		Calcium Superphosphate	
	Measured Value (g/L)	Relative Error (%)	Measured Value (g/L)	Relative Error (%)	Measured Value (g/L)	Relative Error (%)
5.00	5.19	3.80	4.89	2.20	5.31	6.20
10.00	10.16	1.60	9.67	3.30	10.45	4.50
15.00	15.35	2.33	14.78	1.47	14.45	3.67
30.00	29.38	2.07	31.32	4.40	28.99	3.37
35.00	35.78	2.23	37.54	7.26	36.78	5.09
40.00	41.87	4.67	40.78	1.95	42.34	5.85
45.00	45.65	1.44	44.68	0.71	43.75	2.78
50.00	50.65	1.30	49.89	0.22	51.45	2.90
55.00	55.67	1.22	53.67	2.42	55.89	1.16
60.00	60.52	0.87	57.67	3.88	60.56	0.93
65.00	65.18	0.28	65.66	1.02	64.56	0.67
70.00	69.89	0.16	70.89	1.27	70.32	0.46
75.00	75.09	0.12	74.88	0.16	75.16	0.21
90.00	90.21	0.23	89.21	0.88	89.56	0.49

## Data Availability

Data available on request from the authors. The data that support the findings of this study are available from the corresponding author, [Li, J], upon reasonable request.
